# The Effect of Luminance on Depth Perception in Augmented Reality Guided Laparoscopic Surgery

**Published:** 2023-02-23

**Authors:** Athena Reissis, Soojeong Yoo, Matthew J. Clarkson, Stephen Thompson

**Affiliations:** a Wellcome/EPSRC Centre for Interventional and Surgical Science, University College London, United Kingdom; b UCL Interaction Centre, University College London, United Kingdom

**Keywords:** Augmented reality, laparoscopic surgery, image guidance, depth perception, luminance contrast, visualisation

## Abstract

Depth perception is a major issue in surgical augmented reality (AR) with limited research conducted in this scientific area. This study establishes a relationship between luminance and depth perception. This can be used to improve visualisation design for AR overlay in laparoscopic surgery, providing surgeons a more accurate perception of the anatomy intraoperatively. Two experiments were conducted to determine this relationship. First, an online study with 59 participants from the general public, and second, an in-person study with 10 surgeons as participants. We developed 2 open-source software tools utilising SciKit-Surgery libraries to enable these studies and any future research. Our findings demonstrate that the higher the relative luminance, the closer a structure is perceived to the operating camera. Furthermore, the higher the luminance contrast between the two structures, the higher the depth distance perceived. The quantitative results from both experiments are in agreement, indicating that online recruitment of the general public can be helpful in similar studies. An observation made by the surgeons from the in-person study was that the light source used in laparoscopic surgery plays a role in depth perception. This is due to its varying positioning and brightness which could affect the perception of the overlaid AR. We found that luminance directly correlates with depth perception for both surgeons and the general public, regardless of other depth cues. Future research may focus on comparing different colours used in surgical AR and using a mock operating room (OR) with varying light sources and positions.

## 1 Introduction

### 1.1 Background

Using AR in laparoscopic surgery provides great benefits for surgeons by enhancing the visuals of the unseen anatomical structures intraoperatively. This helps to compensate for the loss of haptic feedback and improves surgical precision and accuracy.^1^ AR overlays 3D detailed anatomical models superimposed directly onto the video image of the internal anatomy presented on the 2D monitor captured from a laparoscope.^1^ To create personalised 3D AR simulation models, pre-operative images from CT or MRI scans are first segmented and converted to surface models. These surface models are registered to the intraoperative view, allowing an accurate overlaid image.^1, 2^ This provides real time intraoperative guidance, giving the ability to identify, for example, organs, tumours, vessels and nerves.^3^ This enables reduced loss of blood by understanding how to avoid vessels, supports better intra-operative decision making, and subsequently helps to reduce complications.^3^ Mudgal (2018) states that AR can compensate for the loss in haptic feedback sense while performing laparoscopic surgery by replacing it with an enhancement in the visual sense.^1^ By providing additional information, such as luminance for depth perception, the planning and execution of surgery becomes more precise and accurate.^1, 4^

### 1.2 Depth Perception

Most laparoscopic surgery is performed using a 2D monitor, which means that the visible anatomy and any AR overlaid will have no intrinsic depth cue.^5^ This is a common problem in AR, in both the medical and non-medical fields^5–10^ and there has been little research for this application.^5^ Limited depth perception leads to depth distortion where it is difficult to perceive depths of internal structures from the AR model.^9^ An example of this could be understanding the distance between a patient’s skin and organs^6^ or the distance between a surgical tool and the target organ.^8^ This is important, especially as it allows surgeons to make critical decisions that correspond to the success of the surgical outcome.^11^ To compensate for this issue and improve depth perception on a 2D monitor, monocular depth cues can be used which all have different effects both individually and in combination.^6, 12, 13^ These can be displayed as pictorial or kinematic depth cues such as occlusion, shadow and motion parallax.^6^ Alternatively, depth cues such as colour and luminance can affect how near or far an object is perceived in a virtual scene.^7^ These all provide different visualisation techniques that each have different levels of impact^12^ providing information regarding the distance between an object and the observer.^5^

### 1.3 Luminance

Luminance is a measure of light and corresponds to how bright a human perceives the source.^14^ It can be measured by calculating its relative luminance (*Y*) using the equation


(1) Y=0.2162R+0.7152G+0.0722B.

Equation 1 takes a colour’s RGB code and determines its luminance relative to white, which holds the highest relative luminance.^15^ A bright colour has a high relative luminance and is perceived as closer to the user, whereas a dark colour has a low relative luminance and is perceived as further away from the user.^7^

### 1.4 Luminance Contrast

Luminance contrast is the difference in luminance between colours of surfaces and/or objects and serves as an important depth cue.^16^ This is what causes bright colours to be perceived as physically closer than dark colours; however, this has mostly been researched in relation to a dark background.^7^ Due to the different luminance levels between an object and its background, when the luminance contrast is higher, objects seem further away from the background compared with when the luminance contrast is lower.^7^ Contrast has been shown in previous studies to be very effective and to significantly influence depth perception regardless of other depth cues.^17^ However it has also been found that brighter colours are perceived as further away to the observer when presented on a bright background as opposed to being perceived as closer to the observer when presented on a darker background.^18^ This contradicts the statement that brighter colours are perceived as closer to the observer.

Therefore, since luminance contrast is a highly effective depth cue,^17^ this research focuses on describing its relationship to depth perception in a surgical setting.

### 1.5 Previous Experiments

Heinrich et al., (2021)^6^ conducted an experiment that used four different visualisation techniques to investigate how they affect depth perception. They recruited 21 participants who did not require any prior medical knowledge to complete this study and who reported no colour vision impairments. First was a distance estimation (DE) task in which the distance between a specific target and the phantom torso skin had to be estimated. A scale tool was presented as a reference to make estimating the depth possible. The distance difference between the actual and the participants’ distance was recorded and statistical significance was determined by using a one-way ANOVA test followed by a paired t-test. The next task was a sorting task (S) where the participants had to sort three differently coloured liver segments into the order of nearest and furthest in reference to the liver surface. The error count was recorded for the number of incorrect orders and statistical significance was determined by performing a χ_2_ goodness of fit test.

Outside the surgical field Do et al., (2020)^7^ conducted a forced choice pairwise comparison experiment that tested the effects of different depth cues (shape, fidelity, colour and luminance) with handheld mobile AR on a sphere, cube and bunny model. 16 participants took part in the experiment where none had reported any visual impairments and 13 of them had played video games regularly before. This study took 6 different colours, each in two different luminance levels, one bright and one dark version. For each pairing of these twelve colours (besides comparing a colour with itself), the participant had to select which object they perceived as closer to themselves in reference to a neutral grey background off of their initial impulse. This was conducted online so participants could use their mobile devices which meant that despite COVID-19, people were able to participate. This was done for four objects that had a combination of simple and complex shapes and low and high fidelity. The results showed that dark colours were perceived as further away and bright colours were perceived as closer, which was likely due to the dark background. Colour and luminance interact with fidelity to portray depth and they have a greater effect as fidelity increases, with luminance also showing to have a greater effect than colour on 3D objects.

A review of the literature investigating surgical AR also shows a variety of similar colours that have previously been used to represent different anatomy including tumours and bloods vessels. This is presented in Table 1.

## 2 Research Hypotheses

This research explores the effects that luminance and luminance contrast have on depth perception in a surgical setting for AR using 2D display devices. To address the gap in the literature, we have designed two experiments to test the following hypotheses:

- Hypothesis 1 (H1): The higher the relative luminance level, the closer an object is perceived.
- Hypothesis 2 (H2): The higher the luminance contrast between two objects, the greater the depth distance that is perceived between them.

## 3 Methods

Both the online and in person studies utilised the SciKit-Surgery libraries^22^ to rapidly develop open-source solutions to support our work.

### 3.1 Online Study

For the online study we developed a web application^23^ that allowed us to overlay liver anatomy on a previously acquired image of a liver captured during laparoscopic surgery. The web application allowed us to easily change visualisation parameters (luminance and lighting) via options passed in the URL.

**Figure 1:**
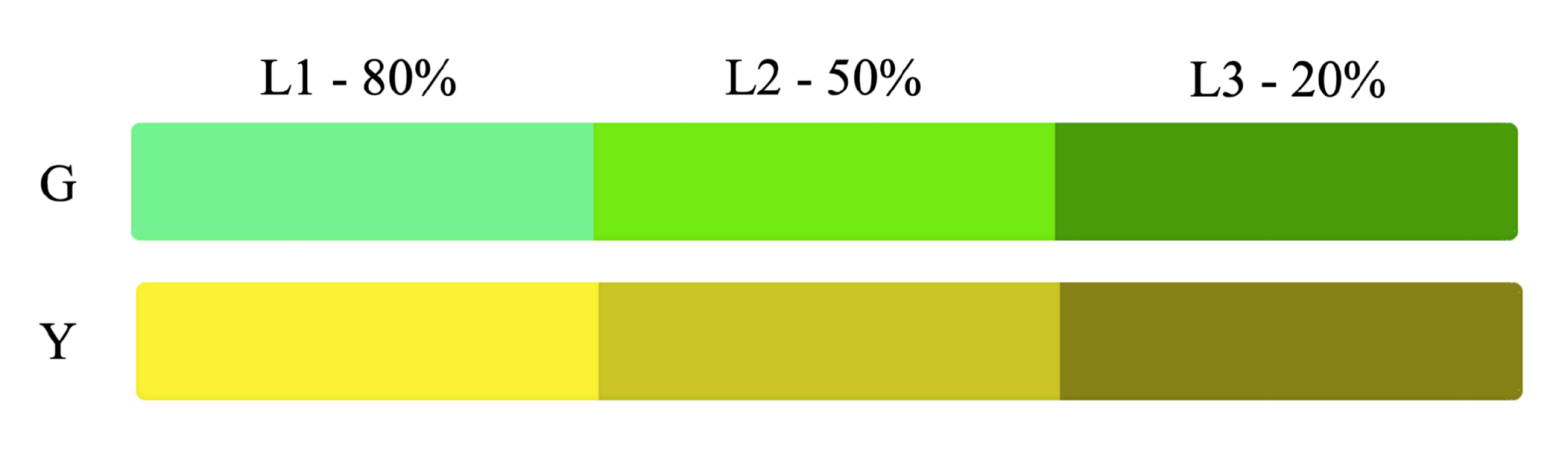
Green (G) and yellow (Y) at three relative luminance levels (L1, L2 & L3). These were the colours used for the tumours in the online study.

We adopted the forced choice pair comparison task that Do et al., (2020)^7^ used to test H1 and also the distance estimation task that Heinrich et al., (2021)^6^ used to test H2. In order to eliminate the impact of other depth cues affecting this experiment, we used two tumours as the objects of interest, keeping them both the same shape and size and leaving them in the same position throughout the study. Blood vessels were also augmented onto the image to give a more anatomical visualisation of the internal structures that would reflect a realistic scenario. The colours and luminance of each tumour were changed for each image and performed both the pair comparison and depth estimation test between these two tumours. From the colours derived in Table 1, we used a green and yellow colour to represent the tumours and use purple for the blood vessels. The colour of the blood vessels remained the same throughout the experiment however we assigned each tumour colour with 3 different relative luminance levels, 20%, 50% and 80%, as seen in Figure.1.

**Figure 2:**
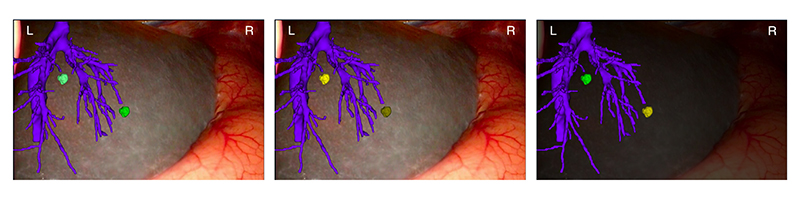
A sample of the images used for the online study, showing changes in tumour luminance and colour together with changes in the background image brightness.

We created 10 images for this study (Table 2). 3 images presented the combination between the three relative luminance levels for the green colour and another 3 images with the yellow colour, all on a background captured directly from a surgical case. Two examples of these are shown in Figure 2. We also compared different colours with the same relative luminance at two levels and also compared altering the opacity of the background to 70% (where 100% would give a completely black background).

This online experiment was performed using Microsoft Forms, with each image being followed by a set of questions. An access link was sent out to the public to gather anonymous data, with 59 responses within the timeframe of the study.

Participants were first introduced to the study and informed consent acquired. Participants were also asked if they had any visual impairments. Then the task was completed with the 10 test images presented one at a time. For each image participants had to perform a forced choice pair comparison task with a binary outcome where they select which tumour they perceived as being closer to the camera, along with the reason why they made this decision.

The next question was the distance estimation task, with set numeric outcomes where participants had to select on a scale of 0cm-10cm the distance in depth they perceived the two tumours to be from each other. These tasks were done for all 10 images and in the same order for all participants. Finally two usability questions were asked at the end of the study.

### 3.2 In-person Study

**Figure 3:**
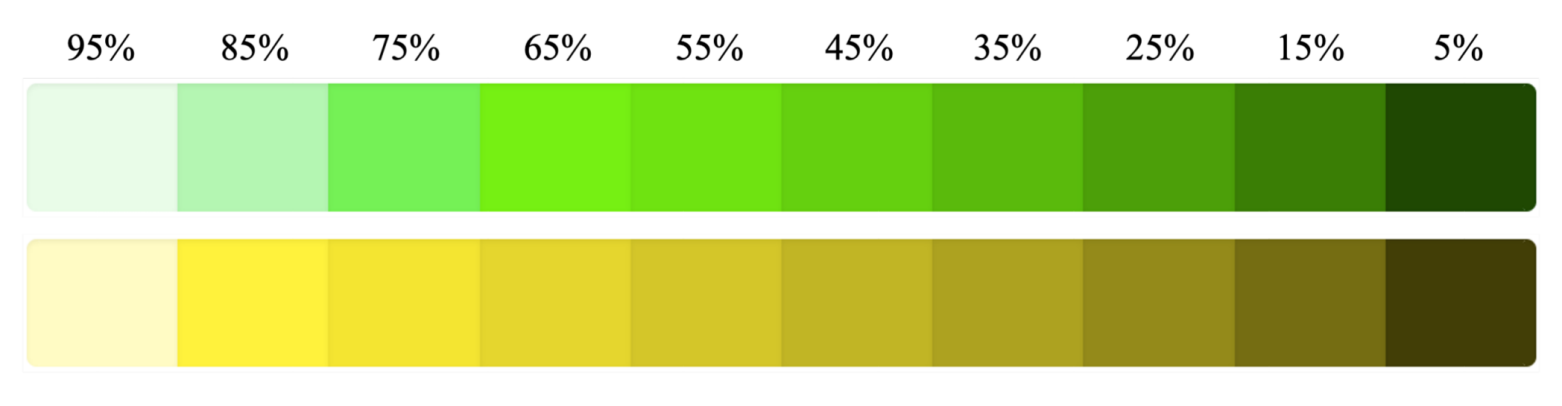
The ten different luminance levels of each colour that is presented in this experiment. The relative luminance values are displayed at the top and correspond to both the green and yellow colours displayed directly below it.

For the in-person study we created a modified version of SciKit-SurgeryBARD^24^ enabling the user to modify the luminance values of each tumour through a touch screen interface in a more realistic scenario of performing surgery. We adopted the same use case as the online study by using a liver structure and overlying two tumours as the objects of interest that remained the same shape, size and positioning, with blood vessels amongst them. However, this time we aimed to incorporate more relative luminance levels and receive information on how surgeons would choose to perceive a depth. Therefore, we designed the experiment to first tell the participants the depth distances of the two tumours with respect to the liver surface, and then for them to select which out of the 10 luminance levels shown in Figure 3 they would use to perceive those depth distances.

**Figure 4:**
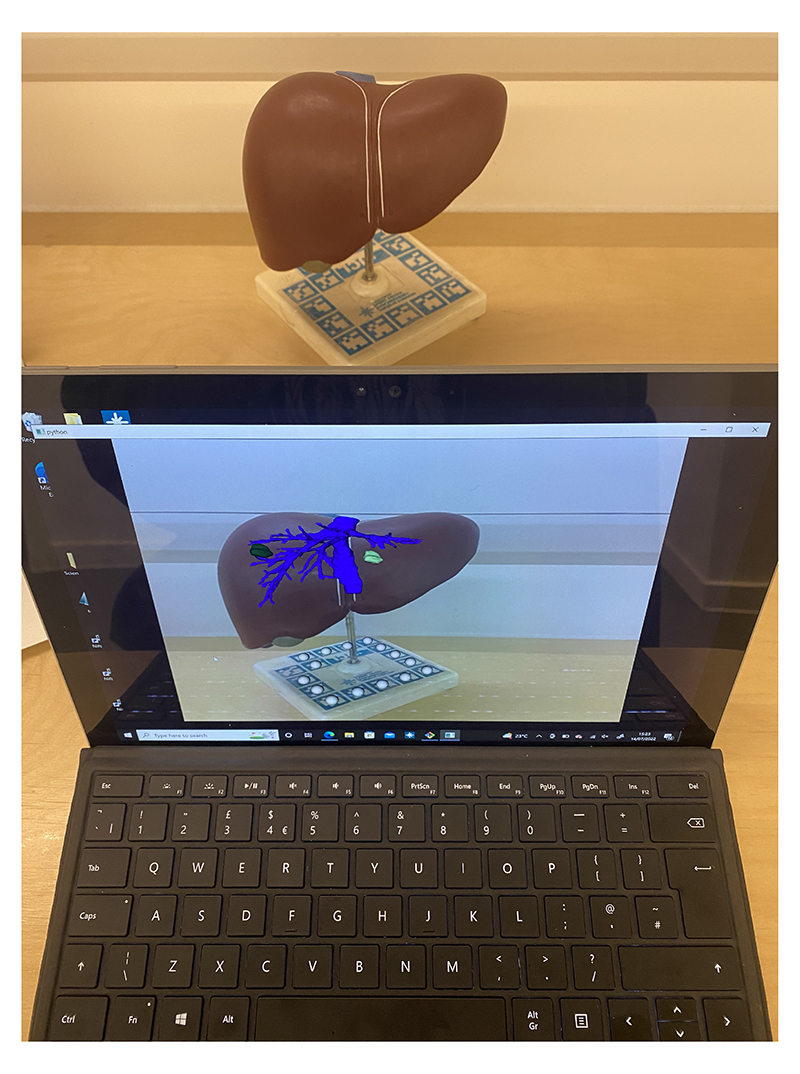
The in-person study utilised an anatomical liver phantom plus see through augmented reality on a tablet computer.

The setup of this study involved a liver model with markers from the ArUco library^25^ at its base and a tablet to overlay the anatomical interactive structures onto the image (Figure 4). We created this transportable setup so we could take the experiment to surgeons themselves making it more convenient to participate and to be able to work around their schedule.

The anonymous study began by receiving informed consent and the participants answering some background questions. During the study we verbally told them the depth distances of each tumour in relation to the liver surface and participants had to alter the tumour’s luminance to represent these distances. To change the luminance of each tumour, participants were able to click on the right and left side of the image to find the preferred visualisation. We used a pair of comparison method taking all possible combinations of 3 different depth distances, 0.5cm, 5cm and 8cm, giving 9 different depth pairings. This was done for both colours and the order of the tasks for each participant was randomised. The task was video recorded collecting qualitative data of their thought process throughout. Finally system usability and evaluation questions were asked about their experience.

10 participants were included in the study, each of whom had a medical degree and at least one year of surgical training, but no prior experience of using AR required. Two participants had used AR in both a medical and non-medical setting before, five participants had never performed laparoscopic surgery and only one reported a visual impairment of a replacement lens for the treatment of cataracts in the left eye.

## 4 Results

The collected data from both the online and the in-person experiments was analysed in relation to both H1 and H2 using Python to create graphs and perform the statistical analysis.

### 4.1 Online Study

#### 4.1.1 H1: The higher the relative luminance level, the closer an object is perceived

Table 3 demonstrates the results that refer to H1. As there were two possible outcomes from this data (’Left’ or ’Right’), the Binomial Test with significance level of 5% is used.

#### 4.1.2 H2: The higher the luminance contrast between two objects, the higher the distance in depth is perceived between each other

**Figure 5:**
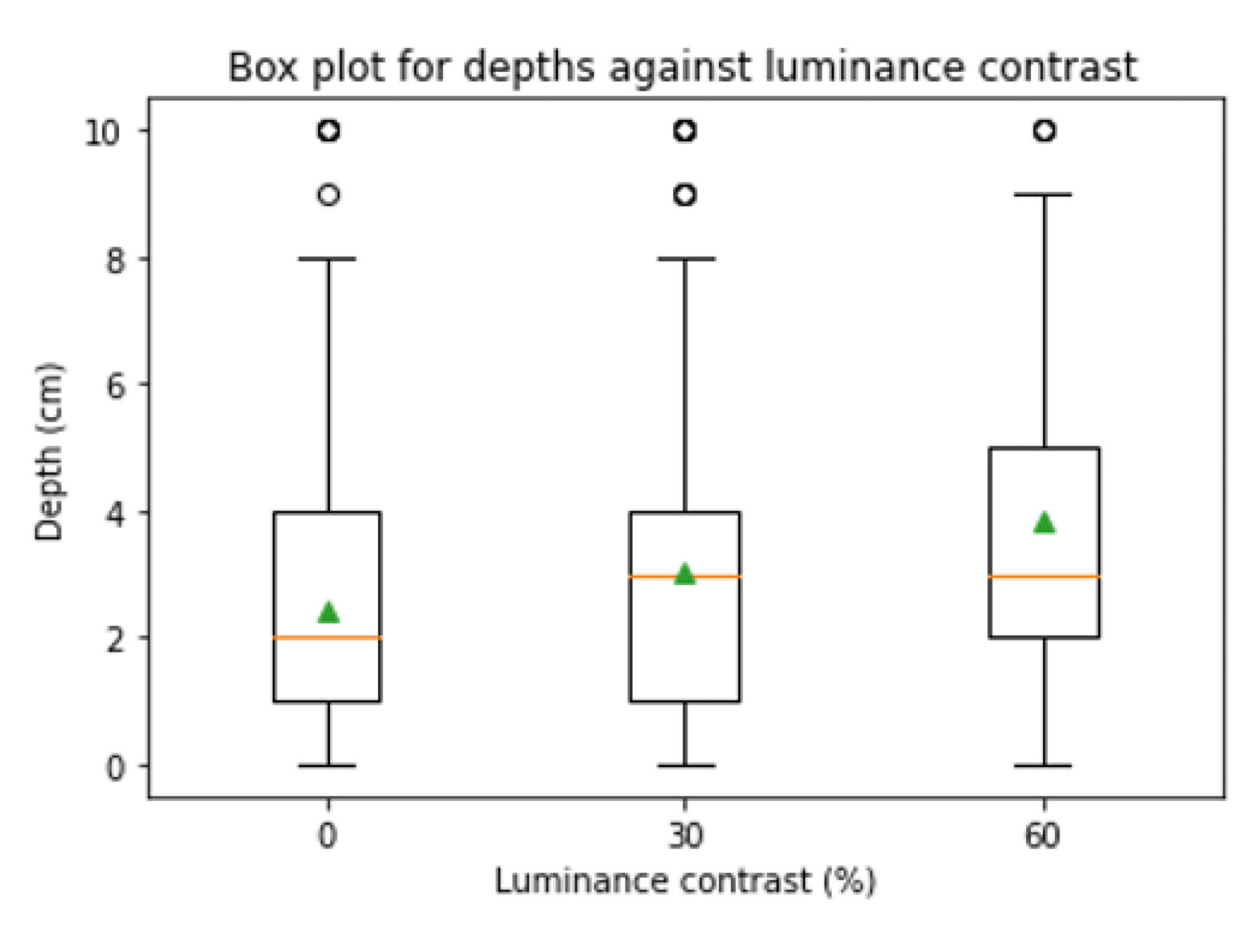
Boxplot showing the depth distances perceived in relation to the luminance contrasts. The green triangles represent the means: 0% - 2.4cm, 30% - 3.0cm, 60% - 3.8cm.

Figure 5 demonstrates the results relating to H2 with the depth distances predicted from the varying luminance contrasts.

Table 4 highlights statistical analysis of the results using a One-Way ANOVA test with a significance level of 5%.

#### 4.1.3 Qualitative results

Table 5 states the most common reasons why participants perceived one tumour to be closer than the other, as collected for each image during the study.

**Figure 6:**
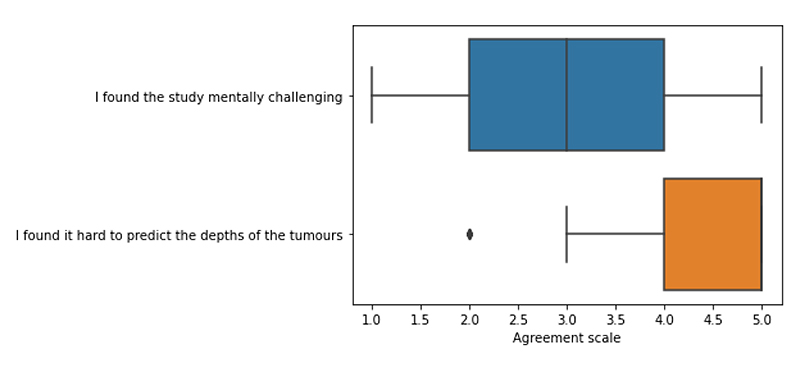
Boxplot presenting the Likert scale results from usability questions. 1 = Disagree and 5 = Agree.

The results from the usability questions displayed in Figure 6 show that participants were neutral about finding the study mentally challenging, but they did find it hard to predict the depths.

General feedback from the participants about the study was that there should have been a ’Same’ or ’Don’t know’ option when selecting which tumour is closer and that it would have been helpful to have a reference for the depth distance measurement to help visualise what 0cm-10cm looked like on their screens.

### 4.2 In-person Study

#### 4.2.1 H1: The higher the relative luminance level, the closer an object is perceived

Results that refer to H1 are presented in Table 6. A Pairwise T-Test was used to compare the luminance levels values of two groups for each task (left and right tumour) with a significance level of 5%.

#### 4.2.2 H2: The higher the luminance contrast between two objects, the greater the depth distance that is perceived between them

**Figure 7:**
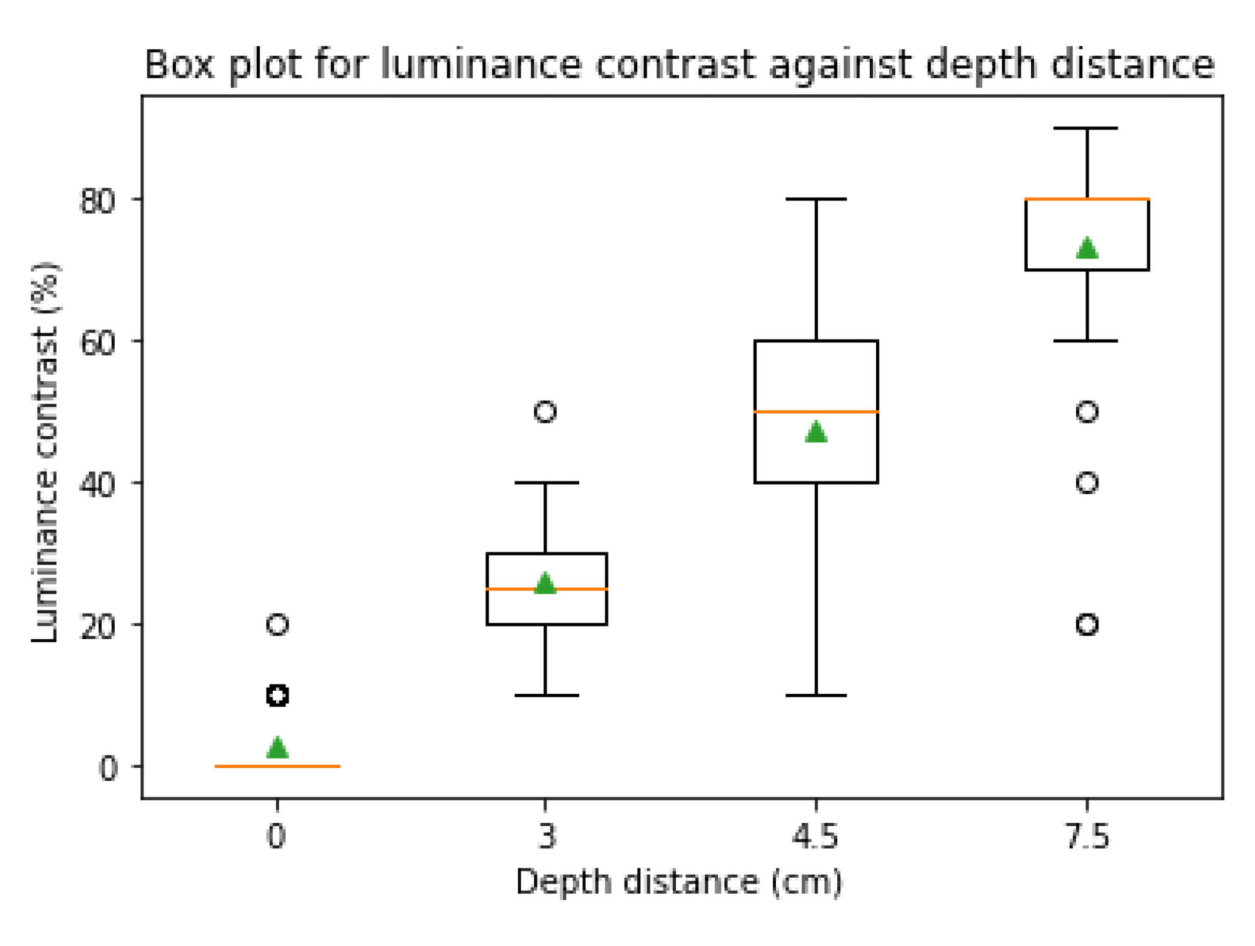
Boxplot showing the luminance contrast selected by the participants to represent certain depth distances. The green triangle represents the means: 0 - 2.5%, 3 - 25.8%, 4.5 - 47.5%, 7.5 - 73.2%.

Figure 7 demonstrates the relationship between depth distance and luminance contrast selected by participants.

Table 7 shows the statistical analysis using a One-Way ANOVA test with a significance level of 5%.

#### 4.2.3 Qualitative results

The confidence level scores for each task on average remained between 3 and 4 (1’Not confident’ and 5’Very confident’). However, from the video recording transcripts, one person commented that they believe their confidence would increase the more they use the system and understand what luminance level corresponds to what depth

**Figure 8:**
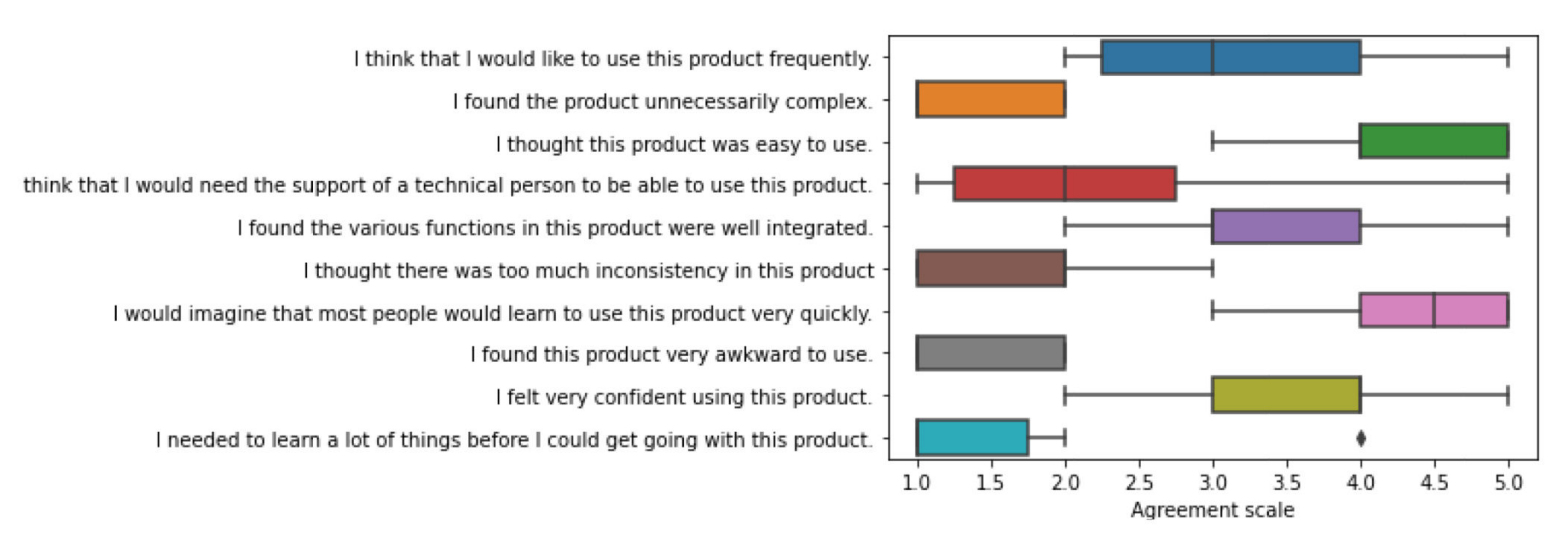
Boxplot presenting the Likert scale results from system usability survey. 1 = Disagree and 5 = Agree.

Table 8 states the most common comments the participants gave in both the evaluation questions and during the task as captured in the transcripts of the video recordings. The results from the system usability survey, as displayed in Figure 8, shows that participants generally found the system simple to use however felt neutral with their confidence using the product and their desire to use the product frequently.

## 5 Discussion

Our first hypothesis (H1) stated that higher relative luminance leads to the user perceiving the object as being closer. This was supported throughout both experiments. For the online study (Table 3), the tumour with the higher luminance was on average always perceived as closer to the camera. During the in-person study (Table 6), the tumour that was said to be closer to the camera was always represented by the participants as the one with the higher relative luminance.

As shown in Table 3, we believe the first test in the online study was not statistically significant due to the participants possibly not being familiar with the anatomical structures and being confused with what exactly the task was. The tasks were unable to be randomised for the online study, so this assumption holds for the same image for each participant. However, having an in-person study with surgeons as participants meant that they already had anatomical knowledge and that giving a demonstration of how to use the system prior to the task allowed for questions and clarifications.

When comparing different colours, we found that at 80% relative luminance the yellow tumour was perceived closer but at a 50% relative luminance the green tumour was perceived as closer for both the normal and dark backgrounds (Table 3). This may suggest that colour as a depth cue could play a role when the luminance levels are the same. Additionally, changing the luminance of the background did not show any significant difference for participants’ perception and answers.

When looking at the position of the tumour, the left tumour was chosen on average to be the closest (all statistically significant). The qualitative data received from both the online and the in-person study (Tables 5 and 8), suggests that this was due to the tumour being within the blood vessel giving the assumption that it was closer, as no luminance contrast was in play. In the in-person study, there was no difference in interpreting the left or right tumour to have the same luminance level for the same depth distances (Table 6).

Our second hypothesis (H2) that a higher luminance contrast between two objects leads to the user perceiving a larger depth distance between them, is also supported by the results from both the online and the in-person experiments. As the luminance contrast increases the depth distance perceived also increases and vice versa (Figures 5 and 7). These results all hold statistical significance (Tables 4 and 7) with the p-values lower than 0.05 along with the increase in F-statistic.

Our results show the average luminance contrast is 34.14% and the average depth distance is 3.46cm. This gives us the relationship of 34.14 × *Luminance_Contrast* = 3.46 × *Depth_Distance* which simplifies down to *Depth_Distance* = 9.867 × *Luminance_Contrast.* We therefore draw the conclusion that:

Depth_Distance≈10×Luminance_Contrast.

Overall, participants perceived a tumour as being closer to the camera mainly if it was brighter than the other tumour (Table 5). Other reasons for this were if they believed that the tumour was bigger in size and also more clear/detailed in structure. The two tumours were the same for every image with size and details not altered throughout the experiment. This shows that luminance and colour could have an effect on how big an object is perceived and how clear its details appear to a user. Another common mention was that a tumour seemed closer as it was higher than the other. This could be affected by the positioning of the camera and light source and the angle at which the image was taken. The liver image from the online study shows as if it were taken at an angle slightly higher than the level of the liver, which could have changed one’s perception thinking that the higher tumour was closer to the camera.

Another common comment made from the in-person study was that the use of more anatomical structures in the AR simulation would have been useful (Table 8). These could also act as a reference point for surgeons by knowing the depths of the standard anatomical structures. Something like this could also add value if this system was used for training purposes. One participant mentioned that with training their confidence levels would increase, perhaps after using the system several times, and that by understanding the meanings of the different colours and luminance levels, surgeons will be more confident with their accuracy. The two participants who stated a ’5 - very confident’ as their confidence level for several tasks both had not used AR before but had performed laparoscopic surgery before. This could indicate that having this experience and familiarity helps with perceiving depth.

A limitation from this study was the number of surgeons we were able to recruit for the in-person study. However from the data presented, the results from surgeons and general public are in agreement, indicating that online recruitment of the general public can be helpful in similar studies. As stated in Table 8, it would have been good to do the experiment in a surgical setting. This would allow the surgeons to interact with surgical tools and provided clarity of the light source, which was also commented on during the in-person study.

As this research focused on only tumours and 2 colours in surgical AR, future work can be done to determine the relationship between more colours, both individually and in combination. This could help to determine if luminance affects each colour in the same way, as well as possibly finding a relationship between a pair of any RGB codes and their perceived depth distance. Another suggestion would be to perform an experiment in a mock OR testing the same hypotheses as this study. This will give surgeons a more realistic working environment providing them also with information regarding the positioning and brightness of the light source needed in laparoscopic surgery.

## 6 Conclusion

This research focused on finding the relationship between luminance and depth perception for AR guided laparoscopic surgery. The purpose of this was to improve surgeons’ depth perception, improve understanding the internal anatomy and subsequently improve surgical outcomes.

Both the online and in-person experiments gave evidence supporting both initial hypotheses, confirming that luminance plays an important role in depth perception in surgical AR.Specifically it was demonstrated that the tumour with the higher relative luminance is perceived as being closer to the camera. Also increasing the luminance contrast increases the perceived depth distance. Thus the relationship between perceived depth distance between two objects (of the same colour) and luminance contrast can be approximated using a standardised equation (Equation 1). This has potential implications for designing future AR software to support surgeons and help improve surgical outcomes for patients undergoing laparoscopic surgery.

## Figures and Tables

**Table 1:** The different colours for tumours and blood vessels used in previous studies for AR in surgery.

Colour references		
Reference	Tumour	Blood vessel
1	Green	Red & Purple
2	NA	Blue & Red & Purple
3	Green	Blue & Red
4	Green & Yellow	Blue
19	Green	Blue & Red
20	Green	Purple
21	Yellow	Blue & Red & Purple

**Table 2:** Final study design that describes the structure of the 10 images for the experiment. N represents normal background, D represents dark background (70% opacity). The colours of the left and right tumour are as stated in Figure 1 with the contrast being the luminance contrast between the two tumours.

Image	Background	Left tumour	Right tumour	Contrast
1	N	G_L1	G_L2	30%
2	N	G_L3	G_L2	30%
3	N	G_L3	G_L1	60%
4	N	Y_L1	G_L1	0%
5	N	G_L2	Y_L2	0%
6	N	Y_L2	Y_L1	30%
7	N	Y_L2	Y_L3	30%
8	N	Y_L1	Y_L3	60%
9	D	Y_L1	G_L1	0%
10	D	G_L2	Y_L2	0%

**Table 3:** Results for perception of which tumour is closer to the camera. Results take the average of all participants. The p-values (3.d.p) were generated from Binominal tests to determine significance.

H1 Results - online study					
Image	Colour	Higher luminance	Results (closest tumour)	p-value	Significant
1	Green	Left	Left	0.117	No
2	Green	Right	Right	1.754e^-10^	Yes
3	Green	Right	Right	1.359e^-9^	Yes
4	Green/Yellow	Same	Left (Yellow)	0.0363	Yes
5	Green/Yellow	Same	Left (Green)	1.698e^-12^	Yes
6	Yellow	Right	Right	5.265e^-8^	Yes
7	Yellow	Left	Left	2.706e^-7^	Yes
8	Yellow	Left	Left	1.907e^-11^	Yes
9	Green/Yellow	Same	Right (Yellow)	0.067	No
10	Green/Yellow	Same	Left (Green)	9.052e^-9^	Yes

**Table 4:** Statistical analysis showing the values from performing ANOVA tests with all combinations of the luminance contrast’s grouped data. If the p-values (3.d.p) falls below 0.05, the test holds significance.

H2 - ANOVA Test (*α* < 0.05)			
Luminance contrasts	F-statistic	p-value	Significant
0, 30, 60	14.973	4.650e^-7^	Yes
0, 30	9.306	0.002	Yes
0, 60	29.178	1.271e^-7^	Yes
30, 60	8.522	0.004	Yes

**Table 5:** Top results regarding the number of times a comment was mentioned when asking participants at each image why they perceived the tumour as closer.

Top results - reasons why tumour is perceived closer	
Reason	Approx mentions
Tumour is lighter/brighter	220
Appears closer	30
Appears bigger	30
Tumour is more clear/detailed	25
Closer to vessel	12
More raised	12

**Table 6:** Results for tumour selection with regard to higher relative luminance level in respect to the known depth distances. The p-values (3.d.p) were generated from t-tests for each task to determine significance

H1 Results - in-person study						
Task (T)	Closer tumour	Green Results (average higher luminance)	Yellow Results (average higher luminance)	p-value (Green)	p-value (Yellow)	Significant
T1	Same	L	R	0.942	0.703	No
T2	L	L	L	3.429e^-6^	6.038e^-5^	Yes
T3	L	L	L	3.464e^-10^	7.590e^-15^	Yes
T4	R	R	R	3.448e^-7^	1.390e^-4^	Yes
T5	Same	R	R	0.749	0.423	No
T6	L	L	L	5.739e^-5^	7.946e^-6^	Yes
T7	R	R	R	5.410e^-10^	1.744e^-16^	Yes
T8	R	R	R	6.633e^-6^	3.957e^-6^	Yes
T9	Same	R	R	0.407	0.749	No

**Table 7:** Statistical analysis showing the values from performing ANOVA tests with all combinations of the depth distances grouped data (0cm, 3cm, 4.5cm & 7.5cm). If the p-values (3.d.p) falls below 0.05, the test holds significance.

H2 - ANOVA Test (*α* < 0.05)			
Depth distance contrast (cm)	F-statistic	p-value	Significant
0, 3, 4.5, 7.5	96.620	1.430e^-68^	Yes
0, 3, 4.5	227.776	2.714e^-44^	Yes
0, 3, 7.5	511.685	2.758e^-64^	Yes
0, 4.5, 7.5	396.117	1.119e^-57^	Yes
3, 4.5, 7.5	107.541	3.133e^-27^	Yes
0, 3	249.173	1.145e^-28^	Yes
0, 4.5	430.050	1.282e^-37^	Yes
0, 7.5	949.251	3.264e^-52^	Yes
3, 4.5	54.554	1.440e^-10^	Yes
3, 7.5	236.940	2.373e^-25^	Yes
4.5, 7.5	49.664	6.403e^-10^	Yes

**Table 8:** Top results for the most mentions of the comments taken from the qualitative data received from both the evaluation questions and the video recording transcripts.

Top qualitative results	
Comment	Approx mentions
Would be good to do experiment in a surgical setting	4
Would be useful for training	3
Would be helpful to have more anatomical structures	3
Would be useful to know where the light source is and its brightness	3
Left tumour seems closer as it is within the vessels	2
